# Development of and adherence to artificial gravity and resistive vibration exercises during 60 days of hypoxic 6° head‐down tilt bed rest: BRAVE study

**DOI:** 10.1113/EP093699

**Published:** 2026-05-28

**Authors:** Jack Fortune, Riccardo G. Sorrentino, Spyridon Zamantzas, Victorien Faivre‐Rampant, Lydia Tsoutsoubi, Adam C. McDonnell, Jason T. Fisher, Sara Podgornik, Leonidas G. Ioannou, Urša Ciuha, Igor B. Mekjavic

**Affiliations:** ^1^ Department of Automatics, Biocybernetics and Robotics Jožef Stefan Institute Ljubljana Slovenia; ^2^ Jožef Stefan International Postgraduate School Ljubljana Slovenia

**Keywords:** artificial gravity, bed rest, countermeasure, horizontal exercise, hypoxia, resistive vibration exercise

## Abstract

Artificial gravity (AG) combined with resistive vibration exercise has been proposed as a multi‐system countermeasure for long‐duration spaceflight; however, its operational feasibility during prolonged unloading remains insufficiently characterised. This study primarily evaluated the feasibility, tolerability, and adherence to a structured resistive vibration exercise protocol delivered either under artificial gravity (AGRVE) or in horizontal loading (HRVE) during 60 days of hypoxic ($F_{IO_{2}}$ = 0.14) 6° head‐down tilt bed rest. Secondary observations included delayed‐onset muscle soreness (DOMS) and changes in eight‐repetition maximum strength (8RM) to inform load progression. Sixteen healthy male participants were allocated to HRVE (*n *= 9) or AGRVE (*n *= 8) and completed near‐daily 30‐min training sessions at light (30% 1RM) and moderate (50% 1RM) intensities. Adherence was high in both groups (HRVE: 98.5%; AGRVE: 96.3%). In AGRVE, early‐session interruptions were primarily due to motion sickness or presyncopal symptoms; motion sickness severity declined significantly across repeated centrifugation exposures (*P* < 0.001), consistent with vestibular habituation. HRVE interruptions were predominantly fatigue‐related. DOMS remained low throughout the intervention (typically 0–3/10). 8RM increased over time in both groups (*P* < 0.001). Progressive resistive vibration exercise can be implemented with high adherence during prolonged hypoxic bed rest, including when combined with AG. Motion sickness represents the principal early operational constraint specific to squat‐based AG exposure but diminishes with repeated sessions.

## Abstract (highlights)

- **What is the central question of this study?**
  Can a structured exercise protocol combining artificial gravity and horizontal resistive vibration be safely implemented with high adherence and tolerability during a prolonged (60‐day) hypoxic head‐down tilt bed rest?
- **What is the main finding and its importance?**
  Artificial gravity and horizontal resistive vibration exercise can be implemented with high adherence during 60 days of hypoxic bed rest, with low muscle soreness and manageable motion sickness that diminishes over time. This is the first long‐duration validation of a progressive artificial gravity‐based protocol and the findings provide essential operational evidence to guide the design of future countermeasures for long‐duration spaceflight.

## 1 INTRODUCTION

The successful return from long‐duration space missions depends fundamentally on the development and implementation of effective in‐flight countermeasures (CM) to mitigate the physiological adaptations induced by prolonged exposure to microgravity, referred to as space adaptation syndrome (SAS) (Demontis et al., 2017; Fernandez‐Gonzalo et al., 2026; Sibonga et al., 2007). These physiological adaptations do not immediately present challenges within microgravity environments. However, they pose a significant risk to an astronaut upon re‐entry into Earth's gravitational field. Following prolonged exposure to microgravity environments, returning to Earth's gravity can exacerbate the likelihood of musculoskeletal injuries (Comfort et al., 2021). Similar consequences are anticipated during the mission to Mars, where the approximately 9‐month journey may impair astronauts’ physical performance upon landing and ultimately compromise the astronauts' health and the successful execution of mission‐critical tasks. Thus, developing CMs to minimise the SAS, notably muscle and bone loss, is crucial. These technologies have undergone significant evolution during human spaceflight, progressing from rudimentary exercise systems like the exerciser 1, during Skylab missions, to sophisticated devices currently utilised aboard the International Space Station (ISS), including the Advanced Resistance Exercise Device (ARED) and the T2 treadmill (Petersen et al., 2016; Tanaka et al., 2017). Despite their sophistication, these devices do not entirely prevent microgravity‐induced physiological adaptations and require substantial daily time commitments. Their partial effectiveness is acceptable for ISS‐duration missions but becomes increasingly inadequate as mission durations extend and gravitational transitions become more demanding.

To date, exercise CMs remain the most efficient method for mitigating microgravity‐associated musculoskeletal deconditioning; however, at present, CMs operate without an actual head‐to‐foot gravitational vector, limiting their capacity to preserve orthostatic tolerance and stimulate mechanotransduction for musculoskeletal preservation. Fortunately, artificial gravity (AG) delivered via a short‐arm human centrifuge (SAHC) has emerged as a promising intervention that can stimulate multiple physiological systems (Clément et al., 2015, 2016; Edmonds et al., 2008; Hoenemann et al., 2023; Symons et al., 2009). However, challenges remain regarding its operational feasibility and the physiological implications associated with chronic AG exposure. Critical issues, such as variable gravitational loading gradients, the presence of a Coriolis force, vestibular disturbances, and human tolerance to high angular velocities and revolutions per minute (rpm), are not yet fully understood and represent key barriers to the practical implementation of AG in future spaceflight scenarios. Although SAHCs are actively under research, spaceflight‐ready designs remain conceptual, and their engineering constraints have not been fully defined. As a result, evidence supporting their optimisation and efficacy as in‐flight exercise platforms is limited. Most existing studies address acute AG exposure, whereas chronic adaptation, crucial for long‐duration missions, remains insufficiently characterised (Duda et al., 2012; Piotrowski et al., 2018; Sorrentino et al., 2024). The recently completed 60‐day BRACE bed‐rest study, which combined AG with cycle ergometry, provides the first emerging data on longer‐term AG‐supported exercise protocols (Hedge et al., 2025). Consequently, further chronic‐exposure studies are required before AG can be established as a viable long‐duration CM.

Head‐down tilt bed rest (BR) studies are widely recognised as the gold standard for simulating the effects of microgravity on human physiology, provoking deconditioning processes similar to those observed in SAS (Hargens & Vico, 2016). Furthermore, BR studies enable the evaluation of novel CM strategies using larger participant cohorts than are feasible during actual spaceflight missions (Belavy et al., 2010; Guinet et al., 2020; Kramer et al., 2021; Rittweger et al., 2006). Within this, horizontal resistance exercise platforms, particularly those incorporating resistive vibration exercise (RVE), have demonstrated beneficial effects on specific aspects of musculoskeletal deconditioning, including attenuation of bone loss and improvements in neuromuscular performance (Armbrecht et al., 2010; Rittweger et al., 2010). Previous BR studies have examined the physiological consequences of environmental modifiers, such as hypoxia, which is expected in future planetary habitats and spacecraft. However, studies involving hypoxic conditions have revealed additional challenges to muscle preservation and increased metabolic demands. The 21‐day PlanHab study, which combined horizontal BR with normobaric hypoxia (∼4000 m altitude), observed reductions in whole‐body fat mass across all groups (−4% to −5%) and a significant reduction in energy intake (Debevec et al., 2014). However, the addition of hypoxia to 21‐day BR did not further impair skeletal muscle oxidative function (Salvadego et al., 2018). Peak oxygen uptake and peak cardiac output declined more under hypoxic BR (∼13.5% versus ∼8.6% reduction) than under normoxic conditions (Keramidas et al., 2016). Hypoxia also prevented the usual suppression of erythropoietin during BR, leading to a greater reduction in plasma volume (Keramidas et al., 2016). Acute hypoxia significantly impairs exercise tolerance and performance. For instance, exposure to hypoxic conditions reduces aerobic capacity, time‐trial endurance performance slows by roughly 16–18% on average, and time‐to‐exhaustion can decline by ∼45% (Deb et al., 2018). When combined with physical inactivity in a BR setting, hypoxia roughly doubles the ${\dot{V}}_{O_{2}\max}$ loss seen with BR alone (Keramidas et al., 2017). In contrast, brief high‐intensity efforts (< 2 min) are largely unaffected by acute hypoxia (Deb et al., 2018), and resistance training under moderate hypoxia can still yield muscle hypertrophy and strength gains comparable to training in normoxic conditions (Benavente et al., 2023). Building on these insights, the 60‐day Bed Rest with Artificial Gravity and Resistive Vibration Exercise (BRAVE) study compared the results of an RVE training programme conducted without and with AG.

This investigation was designed as an exploratory, feasibility‐oriented evaluation of a novel AG‐based exercise CM. Given the technical complexity and absence of long‐duration squat‐based AG data, the primary objective was to determine whether structured RVE, with or without AG, could be delivered safely and with sustained adherence during 60 days of hypoxic BR. Accordingly, adherence, session interruptions, progression of motion sickness (MS) and tolerability were the primary outcomes. Physiological measures, including strength performance tests and muscle soreness, were incorporated to guide load progression and characterise responses, but were not intended to establish comparative efficacy between modalities.

## 2 METHODS

### 2.1 Ethical approval

The experiments were conducted on human participants. All participants provided written informed consent prior to enrolment in the study. The study conformed to the standards set by the latest revision of the *Declaration of Helsinki*, except for registration in a public clinical trial database, as the investigation was conducted as a controlled physiological bed‐rest campaign rather than an interventional clinical trial (Clause 35). The study protocol was reviewed and approved by the Committee for Medical Ethics at the Ministry of Health of the Republic of Slovenia (Approval No. 0120–226/2023/4) and by the European Space Agency (ESA) Medical Board. Ethical approval was granted before the commencement of participant recruitment and data collection. A preliminary study on ambulatory participants (Mekjavic et al., 2025) confirmed the safety and effectiveness of the training programmes over a 2‐week period.

### 2.2 Participant information

Twenty‐six healthy male adults were recruited in accordance with the ESA inclusion criteria (ID‐RCB Number: 2022‐A02074‐39), with 24 participants completing the BR. Each participant received a comprehensive booklet detailing their roles and responsibilities within the study. Participants' inclusion in the study was approved by the BRAVE medical team, who based their decision on the results of a comprehensive medical test, including haematological tests, urine analysis, aerobic fitness tests, body composition assessments and a psychiatric evaluation. Members of the medical team also interviewed participants about their nutritional habits, health status and chronic illnesses, and conducted psychiatric and physical screenings. The final decision regarding inclusion in the study was made during a familiarisation day at the ESA ground‐based facility in Planica (Rateče‐Planica, Slovenia), where participants were introduced to all testing procedures, with a particular emphasis on the exercise protocols. A total of 26 healthy adult males were recruited, all of whom complied with the ESA inclusion criteria (ID‐RCB Number: 2022‐A02074‐39). Participants were allocated to the intervention groups using a pseudo‐random allocation procedure. During the pre‐screening and familiarisation phases conducted prior to entering BR, participant tolerance to AG was observed. In instances where a participant initially assigned to the AG group demonstrated poor tolerance and was unable to complete introductory or familiarisation sessions, the participant was exchanged with an individual from the adjacent CM group who had demonstrated adequate tolerance. As such the participants were divided into three groups (mean ± SD): Control group (*n *= 7); age 32.3 ± 7.4 years, weight 78.4 ± 11.3 kg, height 177.7 ± 4.8 cm; horizontal resistive vibration exercise (HRVE) group (*n* = 9): age 28.8 ± 8.3 years, weight 74.7 ± 8.6 kg, height 180.2 ± 4.6 cm; and artificial gravity with resistive vibration exercise (AGRVE) group (*n* = 8): age 28.0 ± 6.4 years, weight 76.6 ± 7.4 kg, height 181.7 ± 2.9 cm. The present analysis focuses exclusively on the two exercise CM groups (HRVE and AGRVE), as the primary objective was to evaluate protocol implementation, adherence and tolerability rather than comparative efficacy against inactivity. The non‐exercising control group was therefore excluded from this analysis to ensure alignment between study aims and reported outcomes.

### 2.3 BR protocol

Potential CMs for space missions can be validated through BR studies that simulate the unloading of weight‐bearing limbs and the relative inactivity of astronauts in weightlessness. These studies adhere to strict guidelines and standard operating procedures, allowing the results of studies conducted at different facilities to be compared. The BRAVE study was conducted in two experimental campaigns, each involving 12 participants. The first campaign was conducted from September to December 2024, and the second campaign from April to July 2025. Each campaign comprised three phases:

- Basic data collection (BDC) phase. Participant entry into the BDC phase was staggered, with two participants commencing the study each day. In this 15‐day phase, participants were not confined to the facility. Still, they were required to follow a strict schedule, including sleep/wake hours, daily medical/physiological tests and nutrition (five meals/day: breakfast, morning snack, lunch, afternoon snack, dinner). During this phase, participants were also familiarised with the RVE devices and performed familiarisation tests. Exercise training was not conducted in this phase.
- Hypoxic 6° head down tilt BR (6°HDT BR). Following the BDC phase, participants commenced the 60‐day 6°HDT BR. During this period, participants had a daily/weekly schedule of medical examinations and tests. The morning period of each day was reserved for the HRVE and AGRVE exercise training protocols. Since participants in each campaign (*n* = 12) were assigned to the three groups (Control, HRVE and AGRVE), only four participants were trained with HRVE and four with AGRVE. The training sessions for each group were conducted simultaneously by two research teams and scheduled between 09.00 and 13.00 h. Approximately 1 h was allocated to exercise training for each participant, which included transferring to the exercise device, placing sensors for cardiovascular monitoring, recovery and cleanup.
- Recovery phase (R). During the 15‐day recovery period, participants no longer engaged in the exercise programme. This period was devoted to conducting the identical BR standard measurements (BSM) as were conducted during the BDC phase. In addition, participants participated in a rehabilitation programme recommended by ESA (Lambrecht et al., 2017).

**FIGURE 1:**
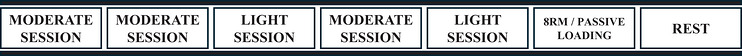
Weekly training schedule implemented for HRVE and AGRVE groups.

During the 60‐day head‐down tilt BR period, participants in both CM groups engaged in 40 scheduled training sessions, in addition to five passive loading sessions for the HRVE group and five passive centrifugation sessions for the AGRVE group, respectively, as well as five eight‐repetition maximum (8RM) strength assessments. Each weekly microcycle consisted of five training days and one rest day, and alternated passive loading and competition day, the latter specifically designed to evaluate participants' progress and inform load prescriptions for the following week (Figure 1). All training sessions were conducted under normobaric hypoxic conditions ($F_{IO_{2}}$: 0.14, ≈4000 m altitude) at a consistent ambient temperature of 21 ± 1°C, with exercise sessions scheduled at the same time daily throughout the study period (i.e., 09.00–13.00 h).

### 2.4 Development and refinement of the BRAVE exercise protocol

**FIGURE 2:**
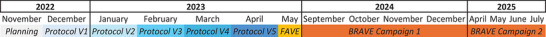
Development timeline of the FAVE (i.e., feasibility of artificial gravity resistive vibration exercise) and BRAVE exercise protocol (Mekjavic et al., 2025).

**FIGURE A1:**
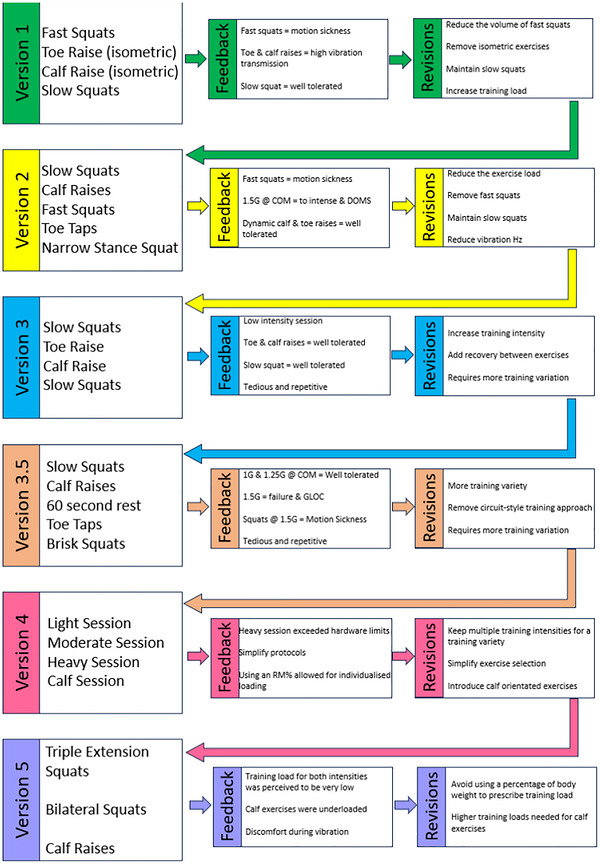
Revision process from original V1 artificial gravity protocol to V5 (FAVE study).

The BRAVE exercise protocol was refined over a year of pilot testing (Protocol versions 1–5) as depicted in Figure 2. The final version of the HRVE and AGRVE protocol was validated following a 2‐week training study in ambulatory participants (Figure 2) to ensure safety, feasibility and adherence to the training programme (Mekjavic et al., 2025). The successful completion of the 2‐week training study led to the implementation of the HRVE and AGRVE training protocols in the 60‐day BR study. The original Version 1 protocol, as detailed in the institutional development report Fortune, et al. (2023) (COBISS.SI‐ID 166280707) (available on request), consisted of bilateral squats (BLS), isometric heel raises, isometric toe raises and fast squats performed at 1 *G* at the centre of mass (COM) with vibration frequencies between 20 and 30 Hz (Appendix Figure A1). Isometric exercises were performed for up to 100 s, and the protocol was repeated in circuit format without inter‐exercise rest. However, prolonged high‐level isometric vibration exposure led to excessive vibration transmission to the head, causing headaches, MS and poor tolerability. These limitations prompted systematic modifications in subsequent protocol versions (i.e., V2–V5) (Figure 2). The primary purpose of Figure 2 is to illustrate the time‐consuming nature of the development of any exercise training programme that includes a new exercise modality. V1–V5 underwent comprehensive modifications across all aspects of the training protocol. These alterations encompassed the elimination and integration of novel exercises, adjustments to loading parameters, modifications to vibration intensities, revisions to recovery intervals and loading protocols, and the introduction of variable training intensities (i.e., light and moderate sessions). Such modifications were progressively implemented across successive protocol versions, aimed at developing a feasible training protocol for the duration of a 60‐day BR, outlined in the report (COBISS.SI‐ID 166280707). In the BRAVE study, the exercise programmes combined several modalities: resistance exercise, vibration and hypoxia. The design of the exercise training protocol drew on methodologies from the first and second Berlin BR studies and incorporated findings from a 2‐week feasibility study (Mekjavic et al., 2025). Minor limitations identified during this study were addressed (i.e., alterations to exercise selection and training progressions), resulting, among other changes, in revised training loads and the addition of single‐leg calf raises (SL‐CR) and proprioception exercises to optimise performance.

### 2.5 Development, testing and validation of a protocol for countermeasure devices

**FIGURE A2:**
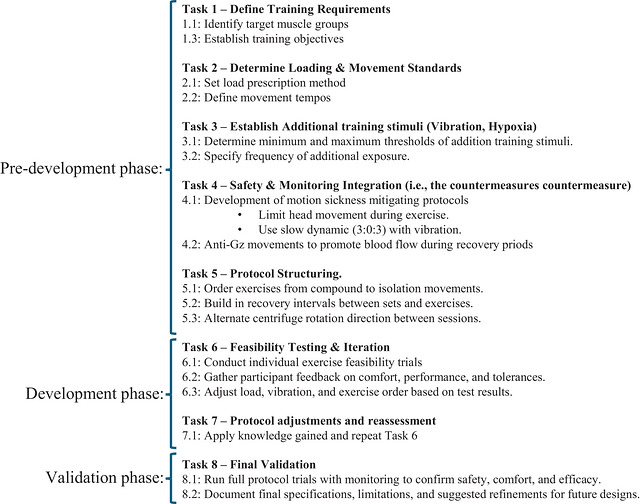
Artificial gravity protocol development and evaluation flow chart.

The development of the prototype exercise device and training programme progressed through three distinct stages, each with a specific aim (Figure A2).

- The pre‐development stage, aiming at defining the mission context, physiological objectives and operational constraints to ensure that subsequent development decisions are grounded in valid requirements.
- The development stage, turning these requirements into a functional and adaptable prototype, integrating safe loading parameters, ergonomic considerations and physiological monitoring.
- The validation phase, qualifying the protocol, confirming its safety, efficacy and operational feasibility, with sustainable training.

#### 2.5.1 Training protocol

The exercise protocols involved three distinct exercises: triple extension squats (TXS), BLS, SL‐CR and bilateral calf raises (BL‐CR) (Tables 1 and 2). Audible cues were delivered through a metronome, accompanied by verbal prompts to help participants maintain proper pacing throughout all exercises. Two other exercises were included in the training programmes, depending on the session's intensity (i.e., light or moderate). Moderate sessions concluded with BL‐CR until failure, while the light training sessions concluded with proprioceptive exercises. All training sessions began with a warm‐up of two sets of eight repetitions of BLS with a 1‐min recovery period between sets at a ground reaction force (GRF) load equivalent to their standing bodyweight. Thereafter, the participants commenced the designated exercise protocol for that day (i.e., light, moderate or competition). All exercises, excluding proprioception exercises, were completed with 20 Hz vibration added and the feet positioned between positions 3 and 4 (i.e., denoted by the 2nd toe placement, which was in line with position 4) on the rotational vibration plate (Galileo vibration plate, Novotech, Pforzheim, Germany). Since the vibration was established due to the side‐alternating movements, the magnitude of the platform's displacement increased from the centre line outwards, with positions 3 and 4 showing displacements of 6 to 8 mm, respectively. To ensure participant safety during all exercise protocols, participants' electrocardiograms (ECGs) and blood pressure were continuously monitored using a five‐lead haemodynamic monitoring system. Arterial oxyhaemoglobin saturation ($S_{pO_{2}}$, %) was also monitored via a pulse oximeter placed on the fingertip (Finapres Medical Systems, Amsterdam, Netherlands). Each exercise (i.e., TXS, BLS and CR) is described below.

**TABLE 1:** Moderate intensity training session.

Exercise	Sets	Reps	Load	Recovery between sets	Recovery between exercises
Triple extension squats (TXS)	4	8	50% of 1RM squat load	60 s	120 s
Bilateral squats (BLS)	4	8		60 s	120 s
Single leg calf raises (SL‐CR)	4 × leg	8		60 s	120 s
Bilateral calf raises (BL‐CR)	1	Failure		N/A	N/A

Repetition volumes (Reps) in the above table represent the prescription when participants entered bed rest without progressions. N/A, not applicable.

**TABLE 2:** Light intensity training sessions.

Exercise	Sets	Reps	Load	Recovery between sets	Recovery between exercises
Triple extension squats (TXS)	4	10	30% of 1RM squat load	60 s	120 s
Bilateral squats (BLS)	4	10		60 s	120 s
Single leg calf raises (SL‐CR)	4 × leg	10		60 s	120 s
Proprioception	1	N/A		N/A	N/A

Repetition volumes (Reps) in the above table represent the prescription when participants entered bed rest without progressions.

##### TXS

**FIGURE 3:**
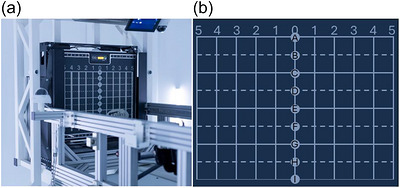
(a) Vibration plate mounted on the SAHC and HRVE device. (b) Participants maintained their feet between positions 3 and 4 on the platform.

Participants were instructed to commence the eccentric phase of the squatting motion from the hips and knees, taking 3 s to transition from a standing position to the lowest squat posture. The concentric phase was again executed over 3 s, moving from the lowest squat position to a complete triple extension of the ankles, knees and hips (including foot plantarflexion). During this movement, vocal cues were provided to ensure correct movement velocities, spine alignment, appropriate foot placement (points 3–4 on the vibration plate) (Figure 3), and to mitigate excessive trunk leaning.

##### BLS

Similar to the TXS, participants were instructed to commence the squatting movement from the hips and knees, maintaining for 3 s the descent from a standing position to the lowest squat posture, with an additional 3 s to return to full knee and hip extension. The verbal prompts utilised for TXS were also applied for BLS.

##### SL‐CR

In the calf raise exercise, participants alternated their feet between each set, keeping the exercising calf at points 3–4 on the vibration plate and placing the resting leg on the black border surrounding the vibration plate. All calf raises were performed at a velocity of 1 s for the concentric phase, followed by a 1‐s hold at the movement's peak, and concluding with a 1‐s eccentric phase (1:1:1).

##### BL‐CR (moderate sessions)

During the calf‐raise exercise, participants stood on the vibration plate with their feet between points 3 and 4, toes pointing forward. They then performed calf‐raise exercises at a 1:1:1 concentric: hold: eccentric velocity (1 s concentric, 1 s hold at the top, 1 s eccentric). Participants performed the BL‐CR until volitional exhaustion or they were instructed to stop if they were unable to maintain sufficient extension and form.

##### Proprioception (light sessions)

Multiple proprioceptive exercises, emphasising coordination and fine motor skills, were incorporated into all light training sessions. These exercises involved participants performing unilateral foot tasks, alternating feet for subsequent proprioceptive challenges. The prescribed activities are detailed as follows:

- Touching toes and heels on designated points on the vibration plate (i.e., ‘touch your right toe/heel on point 5H’) (Figure 3).
- Instructed to draw various shapes of various sizes (i.e., ‘with your right/left foot, draw a small circle inside a large square’).
- Instructed to draw a series of shapes in a specific sequence (i.e., ‘with your right/left foot, draw the following shape in the requested order: circle, square, triangle, square, triangle’).

#### 2.5.2 Participant monitoring and feedback during exercise

Before commencing all exercise activities, participants’ delayed onset muscle soreness (DOMS) was recorded on a scale of 0–10 (i.e., 0 = no muscle pain, 10 = intense muscle pain). Likewise, the participants’ rate of perceived exertion (RPE) was monitored on a scale of 6–20 before and immediately after completing each exercise set (Borg scale) (Borg, 1982). Furthermore, following the completion of each set of exercises, MS was recorded using a motion sickness severity scale (MSSS), which ranged from 0 to 6 (Czeisler et al., 2023). Exercise cessation occurred under the following conditions: excessive fatigue, the participant's wish to stop, and pre‐syncopal symptoms such as tunnel vision, seeing stars, excessive sweating, and high MS levels manifesting as excessive dizziness, nausea and vomiting.

#### 2.5.3 Exercise devices

##### SAHC

**FIGURE 4:**
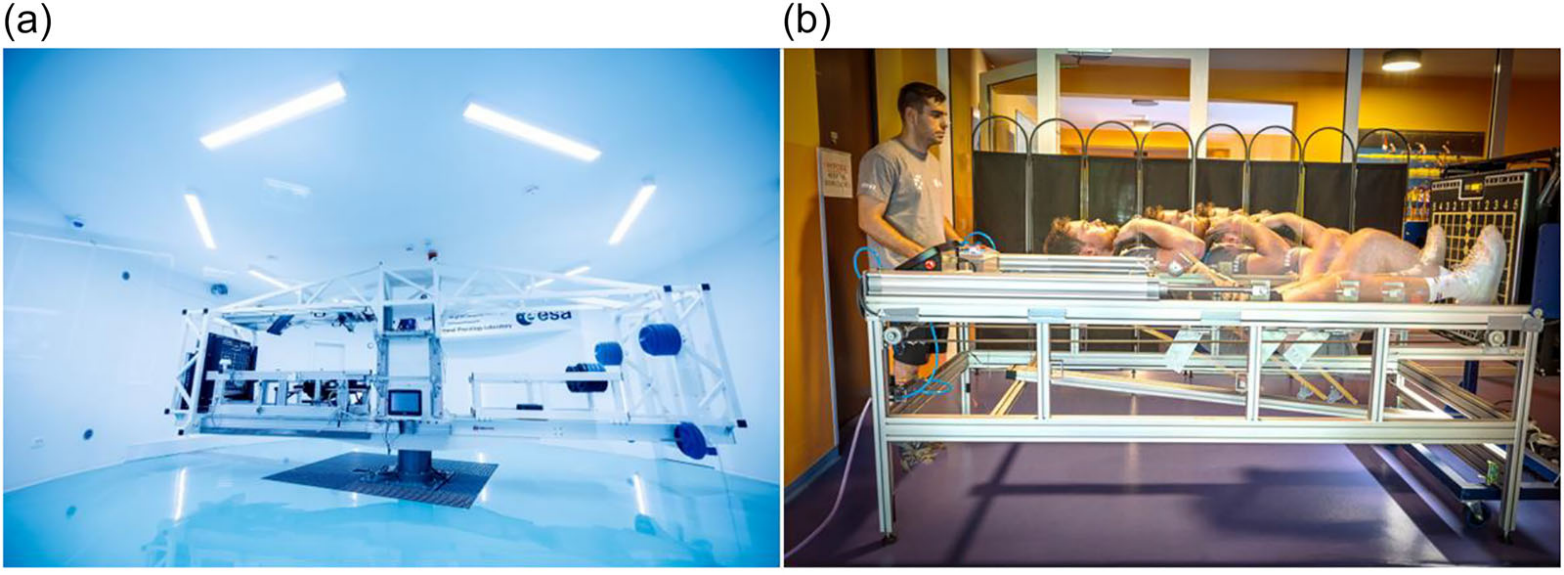
(a) The short arm human centrifuge at ESA's gravitational physiology laboratory, Planica, Slovenia. (b) Horizontal resistive vibration exercise device.

The SAHC has a diameter of 5.64 m (Figure 4a). The system (Redwire, Beveren‐Kruibeke‐Zwijndrecht, Belgium) incorporates a vibration plate (Galileo vibration plate, Novotech, Pforzheim, Germany) mounted at the distal end of one arm, capable of generating vibration frequencies up to 35 Hz. A cradle system (AMST, Ranshofen, Austria) permits movement around two axes, simulating hip and trunk flexion during ambulatory activities. Participants are positioned within the centrifuge and secured using a three‐point harness comprising two hip attachments and one overhead strap, which stabilises the torso to the cradle system. Mechanical loading is generated via angular velocity, producing a centrifugal force vector in the head‐to‐foot (*G_z_*) direction. The centrifuge can reach speeds of up to 32 rpm, corresponding to 4 *G_z_* at the feet. Rotational velocity is adjustable, enabling the operator to modulate training intensity. Due to the short‐arm configuration, a gravitational gradient develops along the longitudinal body axis, resulting in greater mechanical loading at the feet than at the head. Additionally, rotation induces Coriolis forces during head or limb movements, which may stimulate the vestibular system and potentially provoke vestibular disturbances and MS. To mitigate MS, an adjustable mirror is mounted above the participant, allowing visualisation of foot position without head movement during centrifugation. This configuration minimises vestibular stimulation, thereby reducing the incidence of MS.

##### Horizontal resistive vibration exercise device (HRVE)

The HRVE device (Mak d.o.o., Spodnje Senice, Slovenia) is designed to support lower‐body exercises (Figure 4b). The device utilises two pneumatic cylinders that apply controlled force via pistons onto a multi‐axis cradle system, providing a variable load throughout the movement. This configuration closely replicates the forces experienced in AG environments, particularly mirroring the head‐to‐foot gravitational vector encountered during centrifugation. Loading adjustments are achieved by regulating the pressure supplied to the cylinders, expressed as a percentage of their maximum capacity. The device features two operational modes: Manual, where the cylinder pressure remains constant, and Auto, which dynamically adjusts the pressure based on the sledge's position relative to the vibration plate. To mimic the variable load characteristic of SAHC, the pressure at the squat's apex is set lower than at the base, with a linear transition during movement, rising during the eccentric phase and decreasing during the concentric phase.

### 2.6 Training loads

Exercise sessions were performed at two intensities: light and moderate. Although traditional resistance‐training paradigms typically prescribe loads corresponding to 60–80% of one‐repetition maximum (1RM) to optimise hypertrophic adaptations, lower loads were selected for AG‐based exercise. This approach was adopted to avoid undue cardiovascular stress, reduce the likelihood of presyncopal symptoms, and minimise the risk of MS and loss of consciousness, thereby supporting participant safety and maximising training adherence. Based on our preliminary study from the 2‐week Feasibility of Artificial Gravity and Resistive Vibration Exercise study (FAVE) (Mekjavic et al., 2025), training loads for the BRAVE study were increased (FAVE: 25% and 40%; BRAVE: 30% and 50% of 1RM). The increase in training loads was informed by preliminary outcomes from the FAVE study, in which lower intensities were well tolerated but frequently perceived as insufficiently challenging. In response, loading was conservatively progressed while maintaining a primary focus on cardiovascular safety, minimising presyncopal risk, and reducing the incidence of MS under hypoxic conditions. Conventional hypertrophic loading was deemed inappropriate in this context due to the additional vestibular and orthostatic stress imposed by AG. Importantly, BRAVE represents the first long‐duration study to implement AG exercise with a structured progression model and a novel loading method based on GRFs rather than prescribing *G_z_* at a specific anatomical level. Given the limited prior evidence regarding the tolerability of sustained AG exercise, particularly in combination with prolonged head‐down tilt BR and hypoxia, load selection prioritised operational feasibility and participant safety over traditional resistance‐training prescriptions. Participants' 1RM was estimated from an eight‐repetition maximum (8RM) squat test outlined below. The 8RM test was performed biweekly under standardised conditions to ensure consistency in movement execution and recovery. Following each reassessment, 1RM values were recalculated to account for strength adaptations, and training loads were adjusted relative to the updated 1RM to preserve the intended relative intensities (30% and 50% for light and moderate sessions, respectively). Light loads were modified immediately after each 8RM reassessment to facilitate progressive overload within the lower‐intensity training stimulus. In contrast, moderate loads were maintained throughout the first mesocycle (Weeks 1–4) to ensure training stability during early adaptation to AG exposure and to limit abrupt increases in physiological stress. Moderate loads were subsequently recalibrated at the start of the second mesocycle (Week 5), once AG tolerance and session completion had stabilised. For the AGRVE group, training load was assessed at the deepest point of the squat (when there was a 90‐degree bend at the knees) and defined as the combined GRF of body weight (BW) plus the prescribed load (i.e., Light: BW + 30% of 1RM; Moderate: BW + 50% of 1RM). The HRVE device applied a loading pattern distinct from that of the AGRVE system. Under light‐load conditions, it provided constant resistance across the full range of motion, thereby ensuring sufficient force transfer to the vibration platform and preventing excessive foot displacement during exercise, enabling participants to maintain the required foot position. At moderate loads, the device employed a variable loading profile designed to simulate the gravitational loading characteristics of the SAHC.

#### 2.6.1 Familiarisation of both AGRVE and HRVE Sessions 1–3

##### AG familiarisation Session 1: Centrifugation procedures and basic exercise familiarisation

The AGRVE group received a comprehensive orientation covering all components of the SAHC, procedural guidelines, and MS mitigation strategies (e.g., steady breathing, avoiding major head movements, performing slow, controlled movements). Participants were secured with a three‐point harness and equipped with a headset and an instrument to record ECG, blood pressure and $S_{pO_{2}}$. Participants were instructed to recognise MS symptoms (scored 0–6) and to use the Borg Rating of Perceived Exertion (RPE, 6–20). The SAHC was initially accelerated to 8 rpm for 60 s, depending on participants’ verbal comfort. Subsequent incremental increases to 16 rpm were made, with further increases at participants’ request, up to a final rotational speed of 18 rpm. Throughout the session, participants completed two sets each of BL‐CR, BLS, and TXS, with real‐time verbal feedback provided to optimise exercise execution. The total centrifugation duration ranged from 10 to 12 min.

##### AG familiarisation Session 2: Refinement of exercise techniques and introduction to vibration exercise

The spin‐up protocol established during the initial session was repeated. At 18 rpm, participants in the AGRVE group performed two sets of BLS and were subsequently asked about their willingness to increase the rotational speed. Afterwards, the rpm was increased to 20 rpm. At higher rotational speeds, participants were introduced to RVE at 15 Hz. This enabled participants to become familiar with basic movements while experiencing vibration, enhancing their coordination and confidence. Upon demonstrating adequate control and technique, participants performed two sets of each of TXS, BLS and SL‐CR at a vibration frequency of 20 Hz. The session duration was approximately 15–20 min. Additionally, participants were introduced to ‘Anti‐G’ exercises manoeuvres designed to improve cerebral blood flow and during recovery periods (e.g., raise one leg and rest it on the SAHC frame).

##### AG familiarisation Session 3: Establishment of training loads and trial training session

The third familiarisation session was conducted following completion of the final 8RM assessment (see below). Training loads were calculated (i.e., rpm), after which participants performed the prescribed warm‐up, consisting of two sets of eight BLS. Thereafter, participants completed two sets each of TXS, BLS and SL‐CR, all performed at a 20 Hz vibration frequency at their respective moderate training loads. Following each set, participants reported their RPE and MS scores and subsequently engaged in Anti‐G exercises as part of the recovery protocol. The total centrifugation duration ranged from 20 to 25 min.

##### HRVE familiarisation Session 1: Familiarisation with the HRVE device and procedures

The HRVE group received an orientation that covered all components of the HRVE device and its procedural guidelines. Participants were positioned on the HRVE system and instructed on the use of the Borg RPE and MS scales. Initial loading was set at 15% of the total device load (30 kg), during which participants performed two sets of five repetitions for each of the following exercises: TXS, BLS, BL‐CR and SL‐CR. Real‐time verbal feedback was provided to optimise exercise execution. The total familiarisation time was 10 min.

##### HRVE familiarisation Session 2: Refinement of exercise techniques and introduction to vibration exercise

Building on the loading procedures from Session 1, participants performed two sets of BLS at a 15% load. Subsequently, the load was increased to 25% of the device's total capacity (60 kg), at which point participants were introduced to RVE at 15 Hz. Upon demonstrating sufficient control and technique, participants were introduced to the HRVE device's variable load setting, at which point, two sets of TXS, BLS and SL‐CR at a vibration frequency of 20 Hz were performed. The total familiarisation time was 15 min.

##### HRVE familiarisation Session 3: Establishment of training loads and trial training session

As with the AGRVE group, the third familiarisation session was conducted following the final 8RM assessment. After calculating the training loads, participants performed the prescribed warm‐up, consisting of two sets of eight repetitions of BLS. Participants then completed two sets each of TXS, BLS and SL‐CR, all performed at a vibration frequency of 20 Hz at their respective moderate training load. After each set, participants reported their RPE and MS scores. The total familiarisation time was 20–25 min.

##### Familiarisation and test

The 8RM was conducted in accordance with repetition guidelines implemented during the Berlin BR studies to determine the appropriate training loads for participants across both exercise groups (Belavy et al., 2010). To ensure precise execution and participant safety during the maximal strength assessment, participants from both training groups completed two familiarisation sessions using the HRVE device. The first session aimed to familiarise participants with the horizontal squatting technique, emphasising optimal exercise form and proper exercise velocity rhythm. This session commenced with a low training load of 20 kg provided by the HRVE pistons. Any weaknesses in technique were documented and subsequently addressed during a second familiarisation session. The second session aimed to reinforce correct form and further address any remaining issues from the first session at low loads. Once the coaches were satisfied with the participants' performance, a mock 8RM test was conducted, following a warm‐up protocol and three sets of submaximal squatting loads that did not exceed 50 kg. The first 8RM was performed 48 h prior to participants entering the head‐down tilt warm‐up protocol. The warm‐up consisted of one set of eight bodyweight squats with a 1‐min rest, followed by two sets of eight repetitions on the HRVE device at 15% (i.e., 30 kg). Thereafter, during BR, three warm‐up sets at 15% were completed on the HRVE device. Between sets, the participants received a 1‐min recovery period. The squat load was thereafter increased based on the subject's predicted 8RM. The load was increased by 5–10 kg towards the participant's perceived failure point, with a 3‐min recovery period between sets. A final 5‐min rest was provided before the final 8RM attempt. Each participant's 8RM was defined as the final successfully completed set before failure (Table 3). The 1RM load was then calculated using the 8RM value. The test ceased if the participant was unable to complete a repetition successfully, requested to stop verbally, or experienced pain or injury during the test.

**TABLE 3:** Example eight repetition maximum (8RM) test followed by both countermeasure groups.

Eight repetition maximum (8RM) test warm‐up			Eight repetition maximum (8RM) test		
Set	Repetition	Load (kg)	Set	Repetition	Load (kg)
1	8	30	1	8	65
60‐s recovery			180‐s recovery		
2	8	35	2	8	70
60‐s recovery			180‐s recovery		
3	8	35	3	8	80
120‐s recovery			320‐s recovery		
Start test			4	8	85

### 2.7 Progression and regression of exercise

**FIGURE 5:**
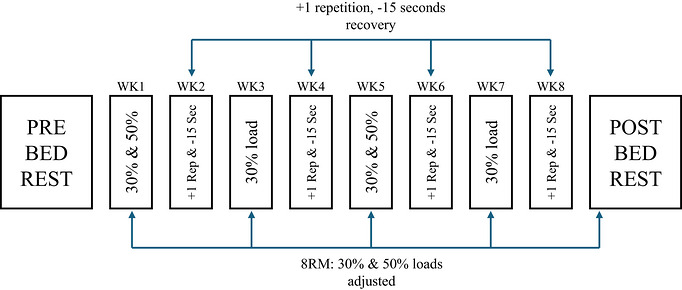
Progression plan implemented for both countermeasure groups during bed rest.

The protocol employed a two‐tiered microcycle exercise progression strategy. Every 2‐week microcycle, participants in both groups followed a standardised progression, in which the number of repetitions per set increased by 1 and the 120 s between‐exercise recovery period was reduced by 15 s (Figure 5). By the end of the intervention, light‐intensity sessions progressed from 10 to 14 repetitions per set, and moderate‐intensity sessions from 8 to 12 repetitions per set, while the recovery interval was reduced from 120 to 60 s over 8 weeks. As aforementioned, strength was assessed biweekly using the 8RM test. If participants maintained their training loads, these were carried over to the subsequent cycle. If the 8RM result indicated an increase, the 1RM was recalculated, and the new loads were applied. Notably, following each 8RM test, light training loads were adjusted accordingly. In contrast, moderate training loads remained unchanged until the start of the second mesocycle (Week 5). Load regression was programmed to occur if participants demonstrated a gradual reduction in squat performance. If this were to occur for the first time, training load was not progressed during the subsequent two training weeks; however, if regressions in 8RM squat assessments continued, training load would be recalculated based on the guidance of the strength and conditioning coaches present during the BRAVE study and on the participant's feedback. Load regression within sessions occurred when participants reported presyncopal symptoms, MS severity ≥4 (MSSS), a high RPE, or fatigue‐related decreases in performance (i.e., an inability to maintain the prescribed technique prior to completion of the target repetition). In such cases, rotational velocity (AGRVE) or pneumatic resistance (HRVE) was reduced incrementally, or the set was terminated.

### 2.8 Statistical analysis

All analyses were performed using JASP (Version 0.18.3, University of Amsterdam, Netherlands). Statistical approaches were selected based on the measurement scale, distributional characteristics and longitudinal repeated‐measures design of each outcome variable. Training adherence (% sessions completed) was analysed using a linear mixed‐effects model with Group, Week and their interaction included as fixed effects and participant entered as a random intercept. Mixed modelling was chosen to appropriately account for repeated observations nested within individuals and to allow inclusion of all available data without requiring complete‐case exclusion. MS severity scores (0–6 scale) were ordinal and demonstrated non‐normal distribution. Therefore, a non‐parametric repeated‐measures analysis was performed using Friedman's test, with Dunn‐adjusted *post hoc* comparisons where appropriate, to avoid violating parametric assumptions. DOMS (0–10 scale) distributions were evaluated using the Shapiro–Wilk test to assess normality. Given the approximately continuous nature of the scale and absence of significant deviation from normality, DOMS was analysed using a linear mixed‐effects model to examine Time and Exercise effects while accounting for repeated measures within participants. For strength outcomes, 8RM performance was analysed using a two‐way repeated‐measures ANOVA with factors for group (HRVE, AGRVE) and time (pre‐ to post‐intervention). Session‐by‐session *post hoc* comparisons were performed to evaluate changes across training sessions. Effect sizes were calculated using Hedges’ *g* and interpreted as small (0.2), moderate (0.5) or large (0.8). All results are presented as means ± SD unless otherwise stated. Statistical significance was accepted at *P* < 0.05.

## 3 RESULTS

### 3.1 Training adherence

**FIGURE 6:**
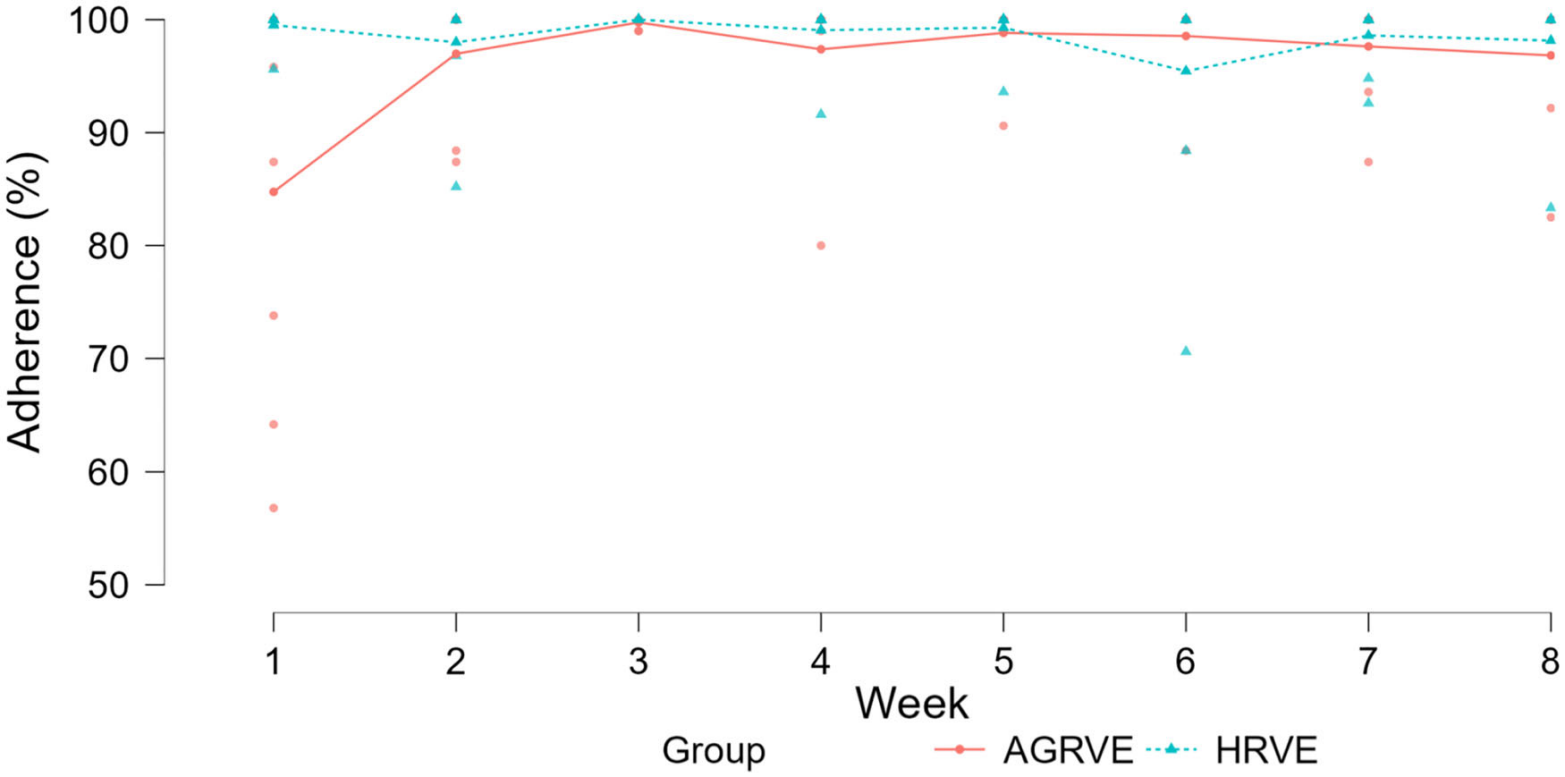
Weekly adherence percentages for the AGRVE and HRVE groups over the intervention period.

A linear mixed‐effects model examined adherence between groups across training weeks (Table 4). The main effect of Group was not significant (*P* = 0.177), indicating that adherence did not differ between the AGRVE and HRVE groups. There was a significant main effect of Week (*P* = 0.001). Group × Week interaction was significant (*P* < 0.001), indicating that adherence trajectories differed between the two intervention groups over time (Figure 6).

**TABLE 4:** Fixed‐effects results from the linear mixed‐effects model assessing the influence of group, week and their interaction on adherence.

Effect	df	*F*	*P*
Group	1, 15.04	2.002	0.177
Week	7, 666.00	3.416	0.001
Group × Week	7, 666.00	4.424	<0.001

Across the AGRVE condition, 28 out of 320 scheduled sessions were incomplete (Table 5). These interruptions occurred predominantly during the first week and were exclusively attributable to MS (n = 10) (Table 6). Despite these early stoppages, a modest increase in training loads was observed throughout the intervention period (Table 7). Likewise, in the HRVE groups, 13 out of 360 sessions were incomplete (Table 8). These instances were primarily associated with excessive fatigue, with a smaller proportion attributed to illness (Table 9). Nevertheless, consistent with the AGRVE condition, training loads demonstrated progressive improvement over time (Table 10).

**TABLE 5:** AGRVE training adherence over the duration of the BRAVE study.

	AG Mesocycle 1			AG Mesocycle 2				
	Week 1	Week 2	Week 3	Week 4	Week 5	Week 6	Week 7	Week 8
Session information								
Total sessions	40	40	40	40	40	40	40	40
Sessions completed	30	38	38	38	38	37	37	36
Incomplete sessions	10	2	2	2	2	3	3	4
Completed (%)	75	95	95	95	95	93	93	90
Set information								
Total sets	760	760	760	760	760	760	760	760
Sets completed	644	737	758	740	751	749	742	731
Incomplete sets	116	23	2	20	9	11	18	29
Completed (%)	84.7	97	99.7	97.4	98.8	98.6	97.6	96.2

**TABLE 6:** Frequency of training cessation in the AGRVE group (Top: Microcycle 1–4, bottom: Microcycle 2).

	AGRVE Mesocycle 1																			
	Microcycle 1					Microcycle 2					Microcycle 3					Microcycle 4				
Day	Mod	Mod	Light	Mod	Light	Mod	Mod	Light	Mod	Light	Mod	Mod	Light	Mod	Light	Mod	Mod	Light	Mod	Light
MS	4	4	—	2	—	—	—	—	2	—	—	1	—	1	—	—	1	—	—	—
Presyncope	—	—	—	—	—	—	—	—	—	—	—	—	—	—	—	—	—	—	—	—
Illness	—	—	—	—	—	—	—	—	—	—	—	—	—	—	—	—	—	—	—	1

	AGRVE Mesocycle 2																			
	Microcycle 5					Microcycle 6					Microcycle 7					Microcycle 8				
Day	Mod	Mod	Light	Mod	Light	Mod	Mod	Light	Mod	Light	Mod	Mod	Light	Mod	Light	Mod	Mod	Light	Mod	Light
MS	—	—	—	—	—	—	—	—	—	—	—	—	—	—	—	1	—	—	—	—
Presyncope	—	1	—	1	—	1	—	1	1	—	1	1	1	—	—	—	1	1	1	—
Illness	—	—	—	—	—	—	—	—	—	—	—	—	—	—	—	—	—	—	—	—

Numbers represent the number of participants who encountered session cessation at a given time point (i.e., 1 = one participant). MS, motion sickness.

**TABLE 7:** AGRVE participants’ individualised load for light (30%) and moderate (50%) sessions expressed as the ground reaction force (GRF, kg) measured on the vibration/force plate.

	AGRVE 1				AGRVE 2				AGRVE 3				AGRVE 4			
Week	1 & 2	3 & 4	5 & 6	7 & 8	1 & 2	3 & 4	5 & 6	7 & 8	1 & 2	3 & 4	5 & 6	7 & 8	1 & 2	3 & 4	5 & 6	7 & 8
30% of 1RM (kg)	35	46	52	57	33	40	43	47	29	38	41	44	31	36	37	41
50% of 1RM (kg)	58	58	86	86	55	55	72	72	48	48	68	68	52	52	62	62

	AGRVE 5				AGRVE 6				AGRVE 7				AGRVE 8			
Week	1 & 2	3 & 4	5 & 6	7 & 8	1 & 2	3 & 4	5 & 6	7 & 8	1 & 2	3 & 4	5 & 6	7 & 8	1 & 2	3 & 4	5 & 6	7 & 8
30% of 1RM (kg)	35	35	38	38	42	42	42	42	48	48	48	48	44	48	48	48
50% of 1RM (kg)	58	58	63	63	70	70	70	70	80	80	80	80	73	73	80	80

**TABLE 8:** RVE training adherence over the duration of the BRAVE study.

	RVE Mesocycle 1				RVE Mesocycle 2			
	Week 1	Week 2	Week 3	Week 4	Week 5	Week 6	Week 7	Week 8
Session information								
Total sessions	45	45	45	45	45	45	45	45
Sessions completed	43	43	45	44	43	42	43	44
Incomplete sessions	2	2	0	1	2	3	2	1
Completed (%)	96	96	100	98	96	93	95	98
Set information								
Total sets	855	855	855	855	855	855	855	855
Sets completed	851	838	855	847	849	816	843	836
Incomplete sets	4	17	0	8	6	39	12	19
Completed (%)	99.5	98	100	99.1	99.3	95.4	98.6	97.8

**TABLE 9:** Frequency of training cessation in the AGRVE group (top: Microcycle 1–4, bottom: Microcycle 2).

	RVE Mesocycle 1																			
	Microcycle 1					Microcycle 2					Microcycle 3					Microcycle 4				
Day	Mod	Mod	Light	Mod	Light	Mod	Mod	Light	Mod	Light	Mod	Mod	Light	Mod	Light	Mod	Mod	Light	Mod	Light
Fatigue	1	1	—	—	—	1	—	—	1	—	—	—	—	—	—	—	—	—	—	1
Illness	—	—	—	—	—	—	—	—	—	—	—	—	—	—	—	—	—	—	—	—

	RVE Mesocycle 2																			
	Microcycle 5					Microcycle 6					Microcycle 7					Microcycle 8				
Day	Mod	Mod	Light	Mod	Light	Mod	Mod	Light	Mod	Light	Mod	Mod	Light	Mod	Light	Mod	Mod	Light	Mod	Light
Fatigue	1	—	—	1	—	1	—	—	1	—	1	—	—	—	—	—	—	—	—	—
Illness	—	—	—	—	—	—	—	1	—	—	—	—	—	—	1	—	—	1	—	—

Numbers represent the number of participants who encountered session cessation at a given time point (i.e., 1 = one participant).

**TABLE 10:** HRVE participants’ individualised load for light (30%) and moderate (50%) sessions expressed as the ground reaction force (GRF, kg) measured on the vibration/force plate.

	HRVE 1				HRVE 2				HRVE 3				HRVE 4			
Week	1 & 2	3 & 4	5 & 6	7 & 8	1 & 2	3 & 4	5 & 6	7 & 8	1 & 2	3 & 4	5 & 6	7 & 8	1 & 2	3 & 4	5 & 6	7 & 8
30% of 1RM (kg)	32	32	33	33	36	54	53	54	32	42	43	44	35	42	42	46
50% of 1RM (kg)	53	53	55	55	60	60	88	88	53	53	72	72	58	58	70	70

	HRVE 5				HRVE 6				HRVE 7				HRVE 8			
Week	1 & 2	3 & 4	5 & 6	7 & 8	1 & 2	3 & 4	5 & 6	7 & 8	1 & 2	3 & 4	5 & 6	7 & 8	1 & 2	3 & 4	5 & 6	7 & 8
30% of 1RM (kg)	39	40	42	42	59	67	61	71	32	37	40	40	42	49	50	50
50% of 1RM (kg)	65	65	70	70	98	98	102	102	53	53	67	67	70	70	83	83

	HRVE 9															
Week	1 & 2	3 & 4	5 & 6	7 & 8												
30% of 1RM (kg)	52	57	57	57												
50% of 1RM (kg)	87	87	95	95												

**FIGURE 7:**
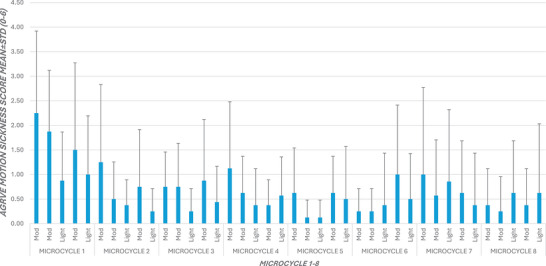
AGRVE motion sickness over the duration of BRAVE.

Motion sickness: MS was non‐normally distributed; therefore, a Friedman's test with a Dunn's multiple‐comparison test was performed (Table 11). Analysis demonstrated a significant effect of time, specifically between the first and final microcycle (Microcycle 1 vs Microcycle 8) (p = 0.036, Hedge's G = 0.99) (Table 11) with week to week compaisons showing small effect sizes following week 1 (Table 12) (Figure 7).

**TABLE 11:** Motion sickness severity across AG sessions (session = chronological AG exposure number, 1–40).

Weeks	*P*	Hedge's *g*
Microcycle 1 vs. Microcycle 8	0.0365	0.99

Hedges' *g* interpreted as small (0.2), moderate (0.5) or large (0.8).

**TABLE 12:** Motion sickness effect size between microcycles (i.e., 1–8).

Motion sickness effect size (Hedge's *g*)						
Week 1 vs. Week 2	Week 2 vs. Week 3	Week 3 vs. Week 4	Week 4 vs. Week 5	Week 5 vs. Week 6	Week 6 vs. Week 7	Week 7 vs. Week 8
0.87	0.02	0.02	0.33	0.12	0.24	0.24

Hedges’ *g* interpreted as small (0.2), moderate (0.5) or large (0.8).

### 3.2 DOMS

**FIGURE 8:**
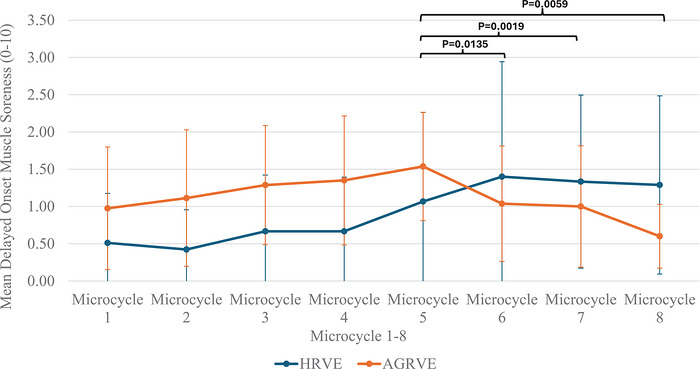
Mean delayed onset muscle soreness reported by both groups.

Shapiro–Wilk testing confirmed that DOMS values did not significantly deviate from normality at any assessment point (all *P* > 0.05), supporting the use of parametric modelling. A Friedman test demonstrated a significant effect of time on DOMS across the 8 weeks, with mean ranks differing significantly between weeks. A mixed‐effects model showed a significant main effect of Time (*F*(3.377, 50.65) = 2.69, *P* = 0.05) and a significant Time × Exercise interaction (*F*(3.377, 50.65) = 4.14, *P* = 0.0083), while the main effect of Exercise was not significant (*F*(1, 15) = 0.37, *P* = 0.55). Matching across repeated measures was significant (χ^2^ = 70.89, *P* < 0.0001), and Tukey‐adjusted *post hoc* comparisons revealed no significant between‐group differences at any individual week. Within‐group *post hoc* analyses revealed no significant pairwise differences in HRVE across weeks after adjustment for multiple comparisons. Comparisons on Microcycle 5 revealed a significant change in DOMS severity within the AGRVE condition across subsequent microcycles. Specifically, DOMS during Microcycle 6 differed significantly from Microcycle 5 (*P* = 0.0135), as did Microcycle 7 (*P* = 0.0019) and Microcycle 8 (*P* = 0.0059). These findings indicate that the elevated soreness observed during Microcycle 5 was followed by a significant reduction in subsequent microcycles (Figure 8). Effect size analysis (Hedges’ *g*) supports this interpretation. In AGRVE, the largest change was observed between Microcycles 5 and 6 (*g* = 0.91), representing a large effect, followed by moderate effects for Microcycle 5 vs. 8 (*g* = 0.62) and Microcycle 5 vs. 7 (*g* = 0.05; small) (Table 13).

**TABLE 13:** Delayed onset muscle soreness effect size between microcycles (i.e., 1–8).

Delayed onset muscle soreness effect size (Hedge's *g*)							
Group	Week 1 vs. Week 2	Week 2 vs. Week 3	Week 3 vs. Week 4	Week 4 vs. Week 5	Week 5 vs. Week 6	Week 6 vs. Week 7	Week 7 vs. Week 8
HRVE	0.15	0.37	0.00	0.40	0.24	0.05	0.04
AGRVE	0.20	0.20	0.08	0.50	0.91	0.05	0.62

Hedges’ *g* interpreted as small (0.2), moderate (0.5) or large (0.8).

### 3.3 Eight repetition maximum (8RM)

**FIGURE 9:**
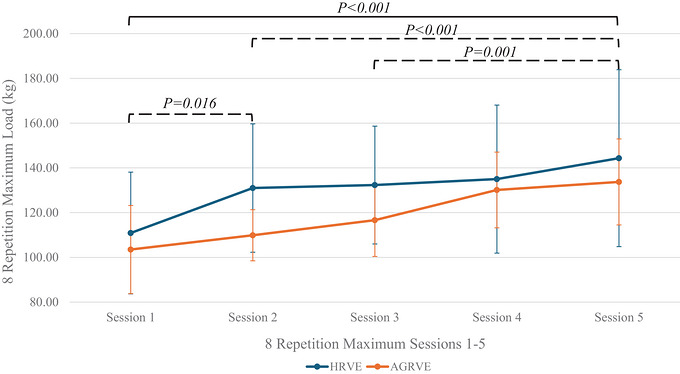
Mean 8 repetition maximum (8RM) values ranging from the first (pre‐HDT) to the final session (final week of HDT).

A two‐way repeated‐measures ANOVA examining the effects of Group (HRVE vs. AGRVE) and Time (Sessions 1–5) revealed no significant Group × Time interaction, indicating that changes in 8RM performance across sessions did not differ between groups. A significant main effect of Time was observed (*P* < 0.001), demonstrating progressive increases in 8RM performance across the intervention period. No significant main effect of Group was detected (*P* > 0.05). Session‐by‐session *post hoc* comparisons revealed a significant improvement between Sessions 1 and 2 (*P* = 0.016, Hedges’ *g* = 0.607). No significant differences were observed between adjacent sessions thereafter (Sessions 2 vs. 3, 3 vs. 4, or 4 vs. 5; all *P* > 0.05). However, performance during Session 5 was significantly greater than during Session 2 (*P* < 0.001, *g* = 0.534) and Session 3 (*P* = 0.001, *g* = 0.348) (Figure 9 and Table 14).

**TABLE 14:** Eight repetition maximum (8RM) effect sizes between microcycles (i.e., 1–8).

	Eight repetition maximum (8RM) effect size (Hedge's *g*)			
Group	Week 1 vs. Week 2	Week 2 vs. Week 3	Week 3 vs. Week 4	Week 4 vs. Week 5
AGRVE	0.4	0.48	0.82	0.19
RVE	0.72	0.05	0.09	0.25

Group	Week 1 vs. Week 5	Week 2 vs. Week 5	Week 3 vs. Week 5	Week 4 vs. Week 5
AGRVE	0.99	0.39	0.36	0.19
RVE	1.55	1.51	0.96	0.25

Hedges’ *g* interpreted as small (0.2), moderate (0.5) or large (0.8).

## 4 DISCUSSION

The principal contribution of this investigation is operational rather than efficacy‐based. The present paper aimed to determine whether structured RVE, performed with or without AG, could be delivered safely and consistently over 60 days of hypoxic BR. Within this framework, adherence, causes of session interruption, progression of MS, and DOMS were the primary outcomes of interest; the present findings offer transparency to inform future protocol refinement and operational implementation.

### 4.1 Training adherence

The HRVE group achieved the highest adherence rate of 98.5%; conversely, the AGRVE group demonstrated an adherence rate of 96.3%. Early AGRVE interruptions (i.e., 28 sessions) were terminated prematurely due to MS in the first mesocycle and presyncope in the second. Of the sessions completed by the HRVE group, 20 instances were recorded in which training was performed at a reduced intensity during SL‐CR exercises due to fatigue or discomfort. Likewise, the AGRVE group had 12 instances in which training load was reduced to accommodate presyncope symptoms. Adherence rates in BRAVE are generally consistent with previous long‐duration bed‐rest studies, such as the second Berlin BR Study, which achieved nearly full compliance with 215 out of 225 prescribed sessions (98%) completed (Belavy et al., 2010). Similarly, the AGBRESA study, which utilised passive centrifugation without exercise, reported adherence rates of approximately 99%, slightly lower than stationary horizontal‐exercise studies such as the Cologne RSL jump protocol (>99 %) (Frett et al., 2020; Kramer et al., 2017). Furthermore, the sister study to BRAVE, the BRACE study, which employed AG combined with cycle ergometry, reported excellent adherence, with only three sessions terminated prematurely. Overall, the AG group attempted 391 of the 392 scheduled sessions, 39 of which were completed at reduced workloads (Hedge et al., 2025).

### 4.2 MS

MS occurred primarily during the first training mesocycle, corresponding to the initial phase of repeated AG exposure, and diminished progressively thereafter, indicating vestibular habituation. Symptoms were most commonly reported during early centrifugation sessions and were frequently associated with squat‐related head movements. No MS events were observed in the HRVE condition, confirming that symptoms were specific to AG exposure. Although all AGRVE participants experienced MS at some stage, session‐terminating severity was largely confined to the early BR period, with both incidence and intensity declining over time. Participants completed three familiarisation sessions prior to BR to attenuate MS risk; however, this preparatory phase did not fully prevent early session termination. Notably, all MS‐related cessations occurred during moderate‐intensity sessions, suggesting that symptom severity was influenced by higher rotational velocities (rpm) and the associated vestibular load. Higher adherence reported in studies such as AGBRESA and BRACE may be attributable to reduced vestibular stimulation, as participants maintained relatively fixed head positions during AG exposure (Clément et al., 2022; Hedge et al., 2025). In contrast, BRAVE required dynamic squat movements under rotation, introducing variable head displacements and changes in rotational speed between warm‐up and training loads. This pattern is consistent with cross‐coupling theory, in which MS arises from vestibular sensory conflict between signals from the semicircular canals and the otolith organs during head movements in a rotating environment. Such discordance between expected and actual vestibular input generates perceptual disorientation and nausea (Bertolini & Straumann, 2016; Paillard et al., 2013). Accordingly, AG protocols that limit head movement during rotation may enhance tolerability and adherence. These findings emphasise the importance of structured familiarisation, gradual progression of rpm, and minimisation of unnecessary head movements during early AG implementation. The rapid improvement in tolerance suggests that MS represents a transient adaptive response rather than a persistent operational limitation for centrifuge‐based CM. It is recommended that future AG habituation protocols incorporate a greater number of familiarisation sessions, exceeding five exposures, to facilitate more robust vestibular adaptation prior to full training implementation. In addition, participants should initially perform AG‐based squatting or resistance exercises at a lower rpm, with gradual, systematic increases over time. Such a progressive approach may attenuate early MS severity, reduce session termination rates, and enhance overall tolerance to centrifuge‐based exercise CMs.

### 4.3 DOMS

DOMS remained low in both groups (0–3/10) throughout the 60‐day intervention, with occasional individual reports reaching 5/10. This modest soreness profile is likely attributable to the submaximal loading strategy. In AGRVE, resistance was prescribed at the deepest squat position, thereby avoiding excessive eccentric strain at extended joint angles; the HRVE protocol was designed to replicate this principle. Although the present protocol does not employ high‐load eccentric training, it is well established that high‐intensity or unaccustomed eccentric contractions are strong drivers of muscle damage, whereas repeated submaximal eccentric sessions rapidly induce protective adaptations that substantially reduce soreness and injury risk (Hody et al., 2019). Furthermore, low‐to‐moderate load eccentric paradigms can elicit meaningful neuromuscular adaptations with lower metabolic and cardiovascular strain, supporting their use in settings where tolerability and safety are paramount (Hody et al., 2019). A transient increase in DOMS was observed during the latter phase of the second mesocycle, coinciding with the first programmed increase in moderate‐intensity loading. This progression represented a programmed increase in mechanical stimulus. The modest rise in AGRVE during microcycle 5 is therefore consistent with an acute response to increased loading. Notably, soreness subsequently declined in microcycles 6–8, suggesting adaptive responses consistent with the repeated‐bout effect, whereby prior exposure to increased loading attenuates perceived soreness in subsequent sessions. In contrast, HRVE demonstrated a slight increase in DOMS between microcycles 5 and 6 while AGRVE values declined. Given that both groups followed identical progression schemes, including repetition increases, biweekly load recalculations and reduced recovery intervals, their mechanical loading environments differed fundamentally. AGRVE loading was generated via centrifugal acceleration, producing a gravitational gradient and joint‐angle‐dependent force variability. HRVE utilised pneumatic resistance to impose consistent, bidirectional loading throughout the movement cycle in light sessions, ensuring stable contact with the vibration plate and likely increasing cumulative mechanical stress. Therefore, between‐group comparisons should be interpreted cautiously, as they reflect distinct mechanical environments rather than direct equivalence across modalities. It is also plausible that by the midpoint of the intervention, participants had developed greater familiarity with the DOMS scale. Early microcycles may reflect conservative or less consistent reporting, whereas the load escalation in microcycle 5 may have provided a clearer perceptual reference point for soreness. Such perceptual changes over time could contribute to small fluctuations in reported DOMS. Collectively, these findings suggest that the observed variations reflect normal adaptation to progressive loading within a training framework.

### 4.4 Repetition maximum

Both the HRVE and AGRVE groups showed comparable improvements in eight‐repetition maximum (8RM) strength during the intervention, indicating a similar rate of strength increase. Significant 8RM gains occurred between the first and second test sessions, highlighting an early‐phase improvement. Initial strength gains are likely attributable to neuromuscular adaptations rather than muscle hypertrophy. Participants became more efficient and comfortable with the equipment, improving neural recruitment and coordination (motor learning and familiarisation). Such early‐phase strength gains are well‐documented to be driven predominantly by neural factors and learning effects, rather than structural changes in muscle, with studies consistently showing that strength can increase substantially in the initial weeks before any measurable muscle growth, owing to increased neural drive and efficiency (Gabriel et al., 2006; Rong et al., 2025). Likewise, repeated strength testing alone yields performance improvements due to exercise familiarisation; multiple test sessions are often required for individuals to express their true maximal strength in a new movement (Soares‐Caldeira et al., 2009). Analyses did not reveal significant differences between adjacent testing sessions (i.e., Sessions 2 vs. 3, 3 vs. 4, or 4 vs. 5); however, significant differences were observed between earlier sessions and Session 5 (i.e., Sessions 1, 2 and 3 vs. Session 5). This pattern is most consistent with a gradual, cumulative improvement across repeated assessments. Stepwise increases between adjacent sessions were relatively small and insufficient to exceed the corrected significance threshold. However, when these incremental changes accumulated over time, the total difference between early and later sessions became detectable.

### 4.5 Additional observations and recommendations

Members of the AGRVE group reported greater difficulty and elevated discomfort levels at the start of each microcycle. The current protocol involves a moderate training session at the outset, which should be replaced with a lighter session when reintroducing participants after consecutive days of training absence. Despite familiarisation, adherence during the first week of AGRVE after BR introduction was lowest. Consequently, this suggests incorporating additional gradual familiarisation sessions, leading to sessions where the protocol is completed in its entirety. In the present study, the first day was designated as a rest day, which likely did not mitigate early plasma loss. Notably, this represents the first AG protocol to incorporate a structured progression and systematic introduction of higher training volumes, providing valuable insights into the practical implementation of progressive overload in an AG environment. Nevertheless, while these findings advance our understanding of AG exercise prescription, a 30‐min exposure is unlikely to fully counteract musculoskeletal deconditioning. Future protocols will therefore need to explore longer or more frequent AG bouts, as well as refined loading strategies, to optimise physiological benefits. Regarding training‐related discomfort and injury, no injuries were reported in the AGRVE group, and discomfort was minimal, primarily mild back or neck strain associated with the SAHC cradle system. In contrast, participants in the HRVE group reported higher levels of discomfort, particularly in the knees and Achilles tendon. Notably, these complaints occurred in individuals with prior issues in those regions, suggesting that the HRVE loading pattern may have exacerbated pre‐existing conditions rather than causing new injuries. Daily physiotherapy mitigated the impact of these symptoms; however, they persisted throughout the study following their first occurrence. The observed patterns offer guidance for future applications of HRVE. Those with pre‐existing musculoskeletal conditions may require a more gradual introduction to loading or modified exercise prescriptions when using HRVE devices to minimise exacerbation of prior injuries.

### 4.6 Conclusion

The BRAVE study demonstrated that both AGRVE and HRVE can be implemented with high adherence during 60 days of hypoxic head‐down tilt BR. Although early AGRVE sessions were limited by MS, symptom severity declined across repeated exposures, consistent with vestibular habituation, whereas HRVE interruptions were primarily fatigue‐related. Notably, only the HRVE group reported excessive fatigue. Both modalities elicited minimal DOMS and were associated with progressive increases in 8RM performance, supporting the tolerability and executability of the prescribed loading scheme. Collectively, these findings provide operational guidance for refining AG protocols, particularly regarding familiarisation procedures, session sequencing and exposure progression. However, while the protocol was feasible and well tolerated, the extent to which the present exposure duration and loading strategy are sufficient to meaningfully mitigate musculoskeletal deconditioning remains uncertain. Ultimately, AG CMs may require longer daily exposure durations, greater cumulative weekly loading, or integration with complementary exercise modalities to optimise physiological protection. The present results, therefore, establish feasibility and provide a structured framework for future efficacy‐driven investigations, rather than confirming the sufficiency of the CM.

## AUTHOR CONTRIBUTIONS

Conception and design of the work: Igor B. Mekjavic, Riccardo G. Sorrentino, Sara Podgornik, Adam C. McDonnell and Urša Ciuha. Acquisition of data for work: Jack Fortune, Riccardo G. Sorrentino, Spyridon Zamantzas, Victorien Faivre‐Rampant, Jason T. Fisher, Lydia Tsoutsoubi, Sara Podgornik, Leonidas G. Ioannou. Analysis and interpretation of data for work: Igor B. Mekjavic, Riccardo G. Sorrentino, Jack Fortune, Jason T. Fisher, Adam C. McDonnell. Acquisition of funding: Igor B. Mekjavic and Urša Ciuha. All authors contributed to the drafting and revision of the work. All authors have read and approved the final version of this manuscript and agree to be accountable for all aspects of the work in ensuring that questions related to the accuracy or integrity of any part of the work are appropriately investigated and resolved. All persons designated as authors qualify for authorship.

## CONFLICT OF INTEREST

None declared.

## Data Availability

The data are available on request.
